# Modes of Antigen Presentation by Lymph Node Stromal Cells and Their Immunological Implications

**DOI:** 10.3389/fimmu.2015.00446

**Published:** 2015-09-08

**Authors:** Sachiko Hirosue, Juan Dubrot

**Affiliations:** ^1^Institute of Bioengineering, École Polytechnique Fédéral de Lausanne, Lausanne, Switzerland; ^2^Department of Pathology and Immunology, Université de Genève, Geneva, Switzerland

**Keywords:** lymph node, stromal cells, antigen presentation, cross-presentation, immunomodulation

## Abstract

Antigen presentation is no longer the exclusive domain of cells of hematopoietic origin. Recent works have demonstrated that lymph node stromal cell (LNSC) populations, such as fibroblastic reticular cells, lymphatic and blood endothelial cells, not only provide a scaffold for lymphocyte interactions but also exhibit active immunomodulatory roles that are critical to mounting and resolving effective immune responses. Importantly, LNSCs possess the ability to present antigens and establish antigen-specific interactions with T cells. One example is the expression of peripheral tissue antigens, which are presented on major histocompatibility complex (MHC)-I molecules with tolerogenic consequences on T cells. Additionally, exogenous antigens, including self and tumor antigens, can be processed and presented on MHC-I complexes, which result in dysfunctional activation of antigen-specific CD8^+^ T cells. While MHC-I is widely expressed on cells of both hematopoietic and non-hematopoietic origins, antigen presentation via MHC-II is more precisely regulated. Nevertheless, LNSCs are capable of endogenously expressing, or alternatively, acquiring MHC-II molecules. Transfer of antigen between LNSC and dendritic cells in both directions has been recently suggested to promote tolerogenic roles of LNSCs on the CD4^+^ T cell compartment. Thus, antigen presentation by LNSCs is thought to be a mechanism that promotes the maintenance of peripheral tolerance as well as generates a pool of diverse antigen-experienced T cells for protective immunity. This review aims to integrate the current and emerging literature to highlight the importance of LNSCs in immune responses, and emphasize their role in antigen trafficking, retention, and presentation.

## Introduction

One of the hallmarks of adaptive immunity is the T cell-antigen-presenting cell (APC) crosstalk that takes place in lymph nodes (LNs). As secondary lymphoid organs, LNs are essential in maintaining tolerance as well as initiating and resolving immune responses (1). LNs constitute particular locations where peripheral tissue environments are sampled in the form of endogenous and exogenous antigens. These processes occur in specific LN regions and are finely controlled by resident stromal cells that promote lymphocyte trafficking and maintain lymphocyte homeostasis.

Lymph node stromal cells (LNSCs) constitute a heterogeneous mixture of non-hematopoietic cells (CD45^−^) of mesenchymal and endothelial origins (2), and have long been appreciated to provide the scaffold on which immune cells encounter antigen (3, 4). Despite their low frequency (<1% of the LN cell count), recent studies have elucidated that LNSCs have active and important roles in shaping the immune response.

The main CD45^−^ LNSC populations can be defined based on their expression of podoplanin (gp38) and PECAM-1 (CD31) by flow cytometry (5–7). They include fibroblastic reticular cells (FRCs: CD31^−^ gp38^+^), lymphatic endothelial cells (LECs: CD31^+^ gp38^+^), blood endothelial cells (BECs: CD31^+^ gp38^−^) (5, 6, 8) and a less studied double negative population (DN: CD31^−^ gp38^−^). Follicular dendritic cells (FDC: CD21^+^, CD35^+^, FDC-M1^+^) constitute an additional subset derived from non-hematopoietic origins (9), which can also express gp38 (5, 10, 11). These LNSC populations help define and organize the structure of the LN as a whole, and because of their distinct anatomic localizations, they can each shape immune responses in complementary ways.

Within LNs, FRCs form an intricate and highly organized reticular network that not only contributes to lymphocyte trafficking but also organizes the lymphocyte populations into different functional zones (12). As the FRC network defines the T cell zone in the cortex, FRCs have been associated with T cell biology. They are a major source of IL7, which is essential for T cell homeostasis (5), and additionally are known to be a source of the chemokines CCL19 and together with LECs, CCL21 (13, 14), which both act on the homing receptor CCR7 on T cells. Moreover, FRCs contribute to the regulation of T cell activation by providing a reticular network that facilitates dendritic cell (DC)–T cell interactions, and subsequent T cell priming (12, 15–17). At the same time, local T cell-derived interferon (IFN)-γ and tumor necrosis factor (TNF)-α act synergistically to induce nitric oxide production by FRCs through the activation of inducible nitric oxide synthase (iNOS) (18–20), thus inhibiting T cell proliferation. More recently, FRCs have been shown to support B cell survival (21).

The lymphatic vascular network, formed by LECs, ensures the transport of antigens from peripheral tissues to local LNs, and then to downstream LNs (22). Within LNs, LECs define the floor and the ceiling of the subcapsular sinus (SCS) and the cortical and medullary sinuses. As lymphatic vessels are the primary route of soluble and APC-carried antigen to the LN, LECs are well-situated to interact directly with lymph-borne antigens, as well as LN-resident and migratory DCs (23), via production of IL7 and CCL21 (24). Under inflammatory conditions, LECs can directly attenuate DC activation through contact-dependent mechanisms (25). Moreover, LECs directly influence T cell activation, in part by dampening activated T cell proliferation through the production of nitric oxide (18, 19). LECs also help direct T cell egress from LNs through secretion of S1P, which is augmented by tenascin-C interactions with α9-integrin on activated LECs (26–28).

BECs line blood vessels that irrigate LNs. The majority of naïve lymphocytes enter LNs from the blood and reach the specific T-cell zones through the specialized high endothelial venules (HEVs) in the cortex (29). HEVs are known to actively promote T cell recruitment and ingress into the LN parenchyma by producing CCL21 (30) and transcytosing CCL19 (31) from the parenchyma.

Specifically located within B cell follicles, FDCs are essential in defining and maintaining the B cell follicular structure, thus shaping humoral immunity (32–34). FDCs have been recently shown to differentiate from marginal reticular cells, which share surface markers with FRCs, yet represent a distinct LNSC subtype located primarily near the SCS and B cell follicles (10). FDCs have a specialized role in maintaining and coordinating B cell responses, including self-reactive B cell deletion (35).

Together, LNSCs create the framework of the LN, with key functional consequences, including guiding peripheral antigens and cells into the LN, and regulating the residence time and interactions of lymphocytes and APCs in the LN (36–38). Immunological roles of LECs and FRCs have been recently reviewed elsewhere (24, 39). Here, we focus on how peripheral antigen sampling and presentation by LNSC may contribute to the overall immune response.

## LNSCs Coordinate Antigen Availability in the LN

Adaptive immunity is a feature of evolved immune systems and relies on the ability of lymphocytes to recognize cognate antigens in the context of a major histocompatibility complex (MHC) (40).

In this regard, there are at least two levels of LNSC participation in antigen presentation. One is the traffic control of antigen distribution, involving the spatial and temporal delivery of LN-infiltrating antigens to specific LN compartments (41). The other resides in the ability of LNSCs themselves to process and present antigens directly to T cells (36).

### Antigen traffic control in the LN

**Figure 1:**
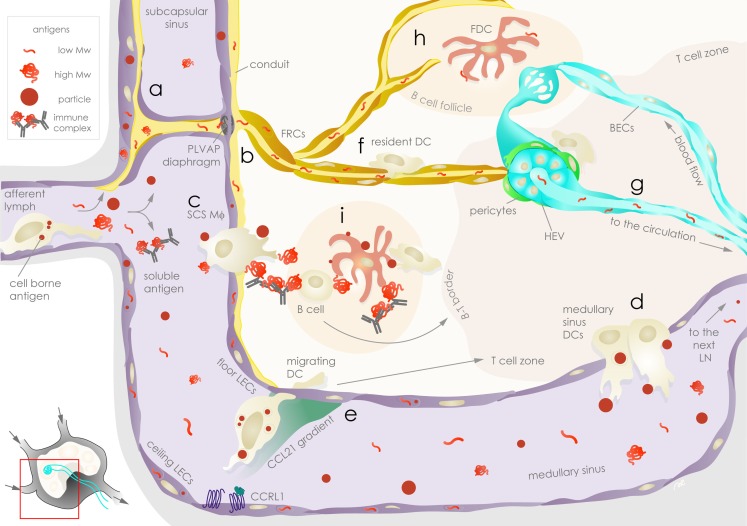
**Exogenous antigen distribution in the lymph node is choreographed by stromal cells**. Lymph-borne peripheral exogenous antigens arrive in the lymph node (LN) via the afferent lymphatic vessels as fast-draining soluble or slow arriving cell-associated forms. *Floor* and *ceiling lymphatic endothelial cells* (*LECs*, violet) are the first stromal cells encountered by the antigens in the subcapsular sinus (*SCS*). Soluble antigens can be taken up and sequestered by *LECs* **(a)**, while the remaining fraction of antigens leave the *SCS*: lower molecular weight antigens are channeled to conduits surrounded by *fibroblastic reticular cells* (*FRCs*, ochre) and are filtered through plasmalemma vesicle-associated protein (*PLVAP*) *diaphragms* on *floor LECs* **(b)**; immune complexes and particles are ferried across the *SCS* by *SCS macrophages* (*Mϕ*) **(c)**. The bulk of soluble antigens not retained or channeled in the above compartments pass through to the *medullary sinus*, where they are sampled by *medullary sinus dendritic cells* (*DCs*) prior to draining to the downstream LN via efferent lymphatic vessels **(d)**. *Floor* and *ceiling LECs* establish chemokine gradients that guide antigen-bearing *migrating DCs* arriving from the periphery by differential expression of atypical chemokine receptors, such as CCRL1, that scavenge CCL21 **(e)**. In the parenchyma, soluble antigens channeled in the conduits are sampled by *FRCs* as well as LN-*resident DCs* **(f)** before they reach the high endothelial venules (*HEVs*, aqua), which are surrounded by *FRCs* or α*7*^+^*pericytes* (green, part of the double negative *DN* stromal population), and enter the circulation where they can be sampled by *blood endothelial cells* (*BECs*, aqua) **(g)**. Conduits also deliver lower molecular weight antigens to follicular dendritic cells (*FDCs*, rose) in the *B cell follicle* **(h)**, while *SCS Mϕ* relay larger antigens to *FDCs* or to *B cells* **(i)**. Antigens on *FDCs* are available for sampling by *DCs* and antigen-specific *B cells* for extended durations. Stromal cells as well as APCs communicate with T cells, contributing to antigen-specific immunological outcomes.

The lymphatic system represents an efficient and sophisticated mode of communication between peripheral tissues and their draining LNs, with the lymph as its medium. The lymph is composed of a mixture of biomolecules, including proteins, peptides, and exogenously derived antigens that reflect the state of the peripheral tissue. Under normal, physiological conditions, lymph has been shown to be significantly enriched, relative to plasma, in intracellular and extracellular matrix proteins and peptides derived from tissue homeostatic turn-over (42). Human lymph has also been shown to carry a more diverse self-peptidome relative to plasma (43). Hence, the lymph fluid appears to represent a comprehensive status report of the peripheral tissues. Following an infection or an inflammatory challenge, exogenous antigens and self-peptides from damaged cells, together with homeostatic components of lymph, enter tissue-draining LNs. This mixture of antigens, including soluble, particulate, and APC-borne forms, is ushered to different anatomic regions of the LN, notably through the interaction with resident LNSCs (Figure 1).

#### LN Entry of Soluble Antigens

Soluble molecules in the lymph enter LNs and are immediately subjected to size-based sorting. Much of our understanding of soluble antigen entry into LNs comes from intravital microscopy studies focused on understanding the generation of humoral immunity (44–47). Upon entry into the SCS, lower molecular weight antigens (≲70 kDa) are immediately channeled to FRC-lined conduits entering the B cell follicles (17), allowing for direct cognate B cell (48) as well as FDC (49) sampling in the follicles, which are important steps toward a humoral response. SCS macrophages (50) and DCs surrounding HEVs (51) also sample antigens. In addition, low molecular weight proteins can also be rapidly sampled by DCs in the LN to elicit T cell responses (52). Meanwhile, a bulk of the remaining, unsampled low molecular weight antigens will end up exiting through HEVs to reach the circulation, or alternatively, through efferent lymphatic vessels, which feed into the next LN (53, 54).

Larger molecules (≳70 kDa) lag behind the small molecular weight antigens by several minutes following LN entry and may take hours to reach cognate B cells (48). This size-dependent distribution and retention time in the LN is in part due to sieve-like diaphragms, composed of plasmalemma vesicle-associated protein (PLVAP) in the SCS LECs (55). PLVAP was originally identified as a BEC-specific molecule that regulates vessel permeability and was recently shown to be important for the formation of fenestrae in liver sinusoidal endothelial cells (LSECs), enabling the regulation of macromolecular transport in the liver (56). In LNs, PLVAP co-localized with the subcapsular and medullary sinus-lining LYVE-1^+^ LECs as well as HEVs (55). PLVAP-deficient mice exhibit an increased permeability of 180 kDa proteins and 500 kDa dextran into the LN parenchyma through the FRC conduits. These observations emphasize the importance of PLVAP in size-based mechanical filtration of antigens in the LN sinus. As a result, larger molecules are generally excluded from the cortex and are forced to pass through the LEC-lined medullary sinuses to drain to the next LN. As a result, these larger molecules pass through an environment rich in LN-resident DCs (sinusoidal DCs) that extend protrusions into the medullary sinus to actively sample the lymph (57). The size-exclusion properties of the LNSC architecture seems to be maintained during inflammatory LN remodeling, as was observed in the case of intradermally administered vaccinia virus (53).

#### LN Trafficking of Particles

Aside from soluble antigens, cell-free antigens can come in the form of particulates, including exosomes, microvesicles, apoptotic bodies, and other membrane-bound particles arising from steady-state and inflammatory conditions (58). Viruses and bacteria, lipid macrostructures, such as alpha-GalCer, and synthetic particles generally occur within a similar size range (59), and are promptly intercepted by the SCS macrophages upon LN entry (57, 60–67), although this capture does not always translate to the development of humoral immunity (63).

Immune complexes, which consist of antigens coated with natural or antigen-specific antibodies and complement proteins, may also behave like “particles” (68). The captured immune complexes are then relayed either directly or alternatively via complement receptor-expressing naïve B cells to FDCs (69, 70).

The remaining unsampled particulates bypass the LN cortex and flow directly to the LN medullary sinus, where they may be captured by LN-resident sinusoidal DCs in a similar fashion as described earlier for the large soluble antigens. Particulate antigens, ranging from 40 nm up to at least 1 μm in diameter were shown to be captured by the sinusoidal DCs (57). This pattern of particles captured in the medullary sinus is consistent with what has been observed for other particulate antigens (63, 71). Furthermore, it has been shown to be essential for the timely generation of both CD4^+^ and CD8^+^ T cell responses to particle-conjugated OVA, while immune responses against soluble antigen developed independently of this mechanism (57).

Thus, while lymphatic transport of larger particulates from the periphery may not be as efficient as that of soluble antigens (72), LNSCs indirectly improve the timeliness of T cell responses by contributing avenues that promote rapid routing of particles in the size range of pathogens to LN-resident sinusoidal DCs.

#### Cell-Associated Antigens in the LN

Antigens may also be carried to the draining LN by peripheral APCs. The trafficking of APCs from the periphery to the LN has been extensively studied (22, 73, 74). In this process, LECs play an important role by promoting APC entry into lymphatic vessels (75–77) and regulating their intra-lymphatic motility (76–78) even when only partial dermal lymph drainage is available (79). In areas of inflammation, inflammatory signals and changes in interstitial fluid drainage into local lymphatic vessels promote DC recruitment and transmigration in several ways. Following binding of immune complexes, DCs become more responsive to recruitment via gradients of CCL19 (80). Inflammatory signals can also increase LEC expression of the adhesion molecules, ICAM-1, VCAM-1, and E-selectin, upregulate CD137 (4-1BB) and CCL21 secretion, and promote CX3CL1 (fractalkine) shedding (81–85). Altogether, these phenotypic changes in LECs and DCs coordinate increased DC transmigration into lymphatic vessels and into draining LNs, in which direct interactions between LECs and DCs are required for efficient DC migration into the LN (86).

While the effects of peripheral lymphatics on DC motility are well documented, the influence of LN-resident LNSC on DC motility is less well known. DC entry into the LN is driven by a chemokine gradient generated by LECs (14). In the subcapsular space, LECs on the afferent side (ceiling LECs) but not on the floor of the capsule (floor LECs) were shown to reduce CCL21 levels due to the expression of atypical chemokine receptor, CCRL1. Such atypical chemokine receptors bind their ligands but fail to signal (87, 88). More importantly, the scavenger function of CCRL1 led to the generation of a localized CCL21 gradient that prompted the entry of DC into the LN parenchyma. In addition to CCRL1, CCL21 has other binding partners, such as podoplanin (89), which may also contribute to the modulation of DC-mediated antigen transport. LNSCs have been shown to partake in intra-LN control of chemokine distribution by other atypical chemokine receptors, such as D6 on afferent LECs and HEVs (90, 91), and Duffy antigen receptor for chemokine transcellular transport (DARC) on HEVs (92). In the LN, binding of CLEC-2 on DCs with gp38 on FRCs, but not LECs, has been demonstrated as a key axis for DC movement (77, 93). Inflammation seems to coordinate CLEC-2 and gp38 expression, which provides an explanation for enhanced interaction between DCs and FRCs (11, 77, 94). By modulating the recruitment of antigen-loaded DCs from peripheral tissues into and within the LN parenchyma, LNSCs help specify the location and duration of cell-borne antigen availability in the LN. Further studies to understand these DC-stromal cell interactions at a molecular level will likely reveal additional insights on the stromal functions in the LN.

### LN antigen retention

Antigen retention is a way of modulating antigen availability during an immune response. Indeed, LNSCs have been known to be safe havens for some pathogens (4, 33). LNSCs are infection targets in some experimental settings, as described for FRCs in a mouse model of persistent lymphocytic choriomeningitis virus (LCMV clone 13) infections (95, 96). The chronicity of the viral burden is sustained in part by upregulation of PD-L1 in infected FRCs that counteract the antiviral CD8^+^ T cell response.

In addition, during acute infections or after vaccination, LECs have been described as antigen reservoirs. Antigen archiving required the induction of LEC proliferation, which, in turn, was an effect of non-cognate T cell proliferation. These observations suggest that T cells can indirectly prolong the persistence of virally derived antigens after the clearance of the virus by promoting antigen archiving function in LECs, which translated to protective memory in the CD8^+^ T cell compartment (97).

Antigens derived from apoptotic cells as well as pathogens are known to be retained in LNs by FDCs (33). FDCs retain antigens on FcγRIIb inhibitory Fc receptors and CD21/CD35 (complement receptors Cr2/Cr1) for presentation to cognate B cells (32, 98, 99). Binding to any of these receptors results in rapid internalization of the immune complexes (100, 101). However, rather than continuing onto a degradation pathway, the engaged complement receptor 2 (Cr2) undergoes periodic recycling and the untampered antigen is therefore made available on the FDC surface over an extended period (100). Immune complexes on FDCs can then be sampled by LN-resident DCs to be presented on MHC-I and II with demonstrated deletion of antigen-specific CD8^+^ T cells (102). Whether other LNSC utilize a similar, recycling, non-degradative compartment remains to be seen. Neither CD21 nor CD35 have been reported to be expressed on other LNSCs (Figure S1 in Supplementary Material), making FDCs unique in this mode of prolonged, intact antigen presentation.

It must be remarked that many studies that discuss antigen uptake and persistence often overlook LNSCs. For example, studies demonstrating particulate antigen capture by CD169^+^ macrophages do not routinely counter-stain for LECs with LYVE-1. However, intradermally injected vesicular stomatitis virus (VSV) results in significant internalization by LYVE-1^+^ cells, regardless of macrophage depletion by clodronate liposomes (61). Similarly, few dynamic imaging studies investigating antigen-specific responses in the LN marked the stroma (12), precluding the opportunity to study the participation of LNSCs. On these grounds, there is a need to clearly define and place LNSC contributions within the current understanding of antigen availability in the LN.

### Structure–function relationships in LNSCs govern antigen availability and influence immune responses

The crucial role of LNSCs in coordinating antigen availability and presentation kinetics of APCs in the LN (3) has been illustrated in a number of inducible knockout models (Table S1 in Supplementary Material). Removing specific LNSC subsets has been achieved in different experimental conditions by expressing the diphtheria toxin (DT) receptor in FDCs (Cd21-Cre) (32), FRCs (Ccl19-Cre, FAP) (103, 104), and LECs (Lyve1-Cre) (105). The absence of any of these LNSC subsets was expectedly sufficient to severely disrupt LN compartmentalization and lymphocyte numbers, leading to an impaired immune response. FDC-depleted LNs cannot retain immune complexes and exhibit deficiencies in germinal center responses (32, 106). Selective depletion of FRCs resulted not only in a deficient T cell response but also correlated with a dramatic reduction of the B cell chemoattractant molecule CXCL12 and the pro-survival factor BAFF, accompanied by follicle destabilization and impaired germinal center formation (103). FRCs appear to regulate the trafficking of mainly naïve lymphocytes as FRC-depleted mice responded to inflammatory challenge with similar numbers of effector T and B cells to normal controls (104). The DT depletion approach was less informative when applied to LECs. In a mouse model where DTR expression is induced in LYVE1^+^ cells, systemic DT administration depleted LYVE1^+^ cells in the intestinal–blood barrier, leading to sepsis (105).

In these depletion studies, lymph and thus antigen flow from the periphery and in the LN is partially maintained. Diminished lymphatic drainage is known to perturb LN B cell organization and humoral immunity (107, 108). However, even when lymphatic drainage is retained, the absence of LNSCs has consequences. Despite the remaining flow of lymph through the ghost FRC conduits, it becomes clear that not only FDCs but also FRCs are indispensable for organizing the boundaries of B cell follicles and the humoral response. This is reflected in the fact that Notch signaling in FRCs has been shown to be important for T follicular helper cell differentiation and germinal center formation (109). This example highlights the synergistic effects of multiple LNSC subtypes and different pathways toward the humoral response, and is likely responsible for regulating the number of B-cell follicles per LN, which otherwise generally scales with the LN size under homeostatic conditions (110).

Another example of synergy between LNSC subsets has been described, where FRCs support the expansion of LECs and BECs by expressing vascular endothelial growth factor (VEGF) and other molecules (8, 111), aided by LN-resident CD11c^+^ cells (112). In addition to supporting the expansion of other LNSC subsets, under inflammatory conditions, the gp38-CLEC-2 signaling axis between FRCs and DCs results in LN structural relaxation by decreasing the contractility of FRCs, ultimately creating space to accommodate increased cell numbers in the LN (113, 114). This pathway is also important in the maintenance of HEV structural integrity as CLEC-2 on platelets can signal through gp38 on FRCs that surround HEVs (115).

In this regard, it is of notable importance that antigen capture, MHC processing, and T cell activation by LN-resident DC are all spatially linked processes (57). While peripheral DC migration to the LN is necessary for a robust immune response, in part by prolonging the time frame of antigen availability within the LN (52, 116), the lymphatic sinus DCs were found to be sufficient to elicit both antigen-specific CD4^+^ and CD8^+^ T cell responses as well as T follicular helper responses, leading to humoral immunity (57). This suggests that antigen drainage and capture occur immediately, triggering an early adaptive immune response and migratory DCs act to amplify the response later upon their arrival to the T cell zone (23). Thus, APC distribution in the LN and their spatial signature (23) affect the kinetics of the immune response. LNSCs are essentially upstream of this process, and in turn, are significantly involved in channeling antigens into the correct subanatomic locations so as to modulate antigen availability to various APC subpopulations.

Taken together, functional lymph flow, antigen sorting, and LN structural integrity maintained by LNSCs are important for the generation of adaptive immune responses. These are manifested in the dynamic LN architecture, which in turn affects the kinetics and time scale of B and T cell activation, proliferation, and polarization as well as feeds back to alter the LN structure and prolong antigen availability.

## Direct Antigen Presentation by LNSCs

There is a growing appreciation that LNSCs not only guide the antigens to APCs but also that they themselves present antigen to educate T cells.

### Brief introduction to antigen presentation

Typically, antigen-presentation pathways are introduced in such a way that intracellular antigens are presented to CD8^+^ T cells as peptide:MHC-I complexes, while extracellular antigens are presented to CD4^+^ T cells as peptide:MHC-II complexes. Presentation of self-antigens by MHC-I on all nucleated cells prevents attack by effector CD8^+^ T cells (117). At the same time, MHC-I presentation allows the presentation of intracellular pathogen-associated epitopes for elimination of infected cells. On the other hand, the processing of extracellular antigens for MHC-II presentation to activate CD4^+^ T cells had been conventionally thought to be restricted to professional APCs. Upon internalization, the extracellular antigens in the trafficking vesicle remain topologically outside while they are processed to generate peptides for MHC-II loading (118, 119). However, neither topological segregation of antigens on the level of the APC nor the exclusivity of certain antigen-presentation pathways to professional APCs captures the full story of MHC-I and MHC-II presentation.

With regards to antigen presentation on MHC-I, it is clear that intracellular antigens from cytoplasmic or intranuclear sources are processed by the proteasome and other cytosolic proteases in the cytoplasm, imported into the endoplasmic reticulum by the transporter associated with antigen-processing (TAP) complex, trimmed, and loaded onto MHC-I. The MHC-I model must also account for exogenous antigens, such as self-antigens draining to the LN under homeostatic conditions, which are presented on MHC-I, the well-known phenomenon of cross-presentation (120). In this process, exogenous antigens leave endocytic vesicles to access the cytoplasmic space for processing and loading onto the MHC-I presenting machinery in a TAP-dependent manner in the endoplasmic reticulum (121–123) or in phagolysosomes (124, 125). Alternatively, exogenous antigens can be loaded onto MHC-I molecules within recycling vesicles in a TAP-independent manner (126, 127). The importance of the cross-presentation pathway can be appreciated in that tumors (aberrant self), and intracellular pathogens (non-self) either lose, or have evolved mechanisms to prevent MHC-I antigen presentation, to evade cytotoxic T lymphocyte (CTL) destruction of tumors, or pathogen-infected host cells, respectively (128, 129). This antigen-processing pathway, previously thought to be the domain of professional APCs, has now been described for other cell types (130).

With regards to MHC-II presentation, phagocytosis and processing of extracellular antigens eventually leads to peptides that are loaded onto MHC-II mainly in MHC-II-enriched multivescicular compartments (131, 132). For epitope peptides to bind to MHC-II, the MHC-II chaperone invariant chain (Ii) must first be cleaved to Class II-associated invariant chain peptide (CLIP). CLIP is then released by the enzyme H2-M to bind the MHC-II epitope peptides for the peptide:MHC-II complex to finally be available on the APC cell surface (133). Interestingly, these proteins involved in MHC-II presentation are not completely absent from LNSCs on the transcription level (8, 134, 135) (Figure S2 in Supplementary Material).

In addition, MHC-II can be loaded with intracellular antigens as suggested by the analysis of MHC-binding peptide repertoire in the thymus (136, 137). Autophagy and endocytosis of apoptotic material can introduce intracellular antigens to the MHC-II pathway (136). Again, existence of viral evasion mechanisms suggests the importance of this pathway (138).

Other intracellular pathways are shared for both MHC-I and -II loading, such as the gamma-interferon-inducible lysosomal thiolreductase (GILT) enzymes in DCs (139), which are thought to aid in antigen translocation into the cytosol (MHC-I) as well as exposure of buried epitopes (MHC-II) (140).

These observations demonstrate that antigen presentation is not limited by the topological origins of the antigen and that antigen presentation pathways previously thought to be restricted to professional APCs can be active in other cell types. As such, both self- and non-self-antigens can be presented on MHC-I and MHC-II molecules. The antigenic self-peptide repertoire is highly plastic (141), and may be changed under inflammatory conditions with the induction of the immunoproteasome (142, 143) or altered MHC trafficking in activated DCs (133). Finally, antigen-presentation capacity is variable in efficiency and duration as well as environmentally modulated in different DC subsets (144). This opens the possibility for antigen presentation by LNSCs to partake in the modulation of the immune response.

### MHC-I presentation by LNSCs

#### MHC-I Expression in LNSCs

Nearly all nucleated cells constitutively express MHC-I, LNSCs being no exception. The MHC-I complex at the cell surface consists of a polymorphic MHC-I together with a non-polymorphic β2 microglobulin (β2M), both of which are regulated by a specific transactivator NLRC5 (145). MHC-I expression can be induced in response to IFN-γ through the IFN-stimulated response element. This upregulation of MHC-I was observed when LNSCs were in the presence of toll-like receptor (TLR) agonists, or engaged in an antigen-specific interaction with T cells (146–148). Along with MHC-I expression, NLRC5 induces TAP1 and LMP2 (proteasome subunit β type 9), coordinating the expression of some main proteins involved in the cytoplasmic antigen-processing and MHC-I-presentation pathways.

#### Self-Antigen Expression in LNSCs

Peripheral tissue antigens (PTAs) can be promiscuously expressed in the thymus in medullary thymic epithelial cells for the purpose of negative selection of T cells in central tolerance (149). However, some self-reactive T cells remain in the periphery (150, 151). Tissue draining, but also non-draining, LN FRCs, LECs, and BECs have been described to express PTA repertoires, including both endogenous PTAs as well as transgenically introduced PTAs (146, 152–155). Interestingly, these repertoires are not overlapping between the LNSC subsets (146, 156), likely attributable to differential expression of transcription factors driving PTA expression, such as Autoimmune regulator (Aire) (146, 154) and deformed epidermal autoregulatory factor 1 (Deaf-1) characterized in the pancreatic LN (157). Within a LNSC subset, PTA expression can further be localized subanatomically (156). For example, in the LN, the PTA Tyr369 is presented only by the medullary LECs as evidenced by antigen-specific CD8^+^ T cell proliferation, when LN LECs were separated into subcapsular, medullary, and cortical LECs based on the differential expression of MAdCAM-1 and PD-L1 (156). The intracellular mechanisms of PTA MHC-I presentation have not been addressed in any of these studies, though it is assumed to be the same as with any endogenously expressed intracellular antigen.

The PTA expression pattern in the mouse suggests that the LN is particular in the presentation of antigens by stromal cells. In the spleen, another important secondary lymphoid organ, stromal cells did not express detectable PTAs compared to LNSCs (152, 153). Moreover, the same stromal cell types in the periphery express fewer PTAs than those in the LN. For example, unlike their LN counterparts, LECs isolated from the diaphragm and colon cannot present the PTA Tyrosinase to antigen-specific T cells (156).

Whether there exist overarching drivers or triggers for the LN-restricted expression of PTA by LNSCs, and how this is subdivided into specific cell types in subanatomic locations, has not yet been explored.

#### Exogenous Antigen Cross-Presentation in LNSCs

As polarized cells with active endocytotic and transcytotic pathways, LNSCs actively take up exogenous molecules and some subsets process antigens for cross-presentation and cross-priming of antigen-specific CD8^+^ T cells. This was confirmed in several models using OVA as a model exogenous antigen, as detected by the 25d1.16 antibody, which recognizes the MHC-I epitope of OVA complexed with MHC-I, SIINFEKL:MHC-I, on APCs. OVA derived from OVA-expressing B16F10 tumors was found to be displayed on MHC-I in LECs in the tumor and the draining LN (158). Similarly, SIINFEKL:MHC-I complexes were confirmed on LECs incubated with synthetic nanoparticles bearing OVA-derived long peptides *in vitro* (148, 158). In these studies, OVA-loaded primary LN LECs were shown to be capable of cross-priming OT-I CD8^+^ T cells in a TAP1-dependent manner (148).

For LN FRCs, FDCs and BECs, on the other hand, no intracellular antigen-processing pathways have been described. However, cross-presentation by BECs has been described in other organs. Primary cultures of murine aortic BECs have been shown to cross-present exogenous male antigen to a T cell hybridoma cell line MHH, specific for the MHC-I (D^b^) restricted male antigen HY*Uty* (159). LSECs also present exogenous antigen in a TAP1-dependent manner (130, 160, 161). This suggests that LN BECs may also cross-present exogenous antigens on MHC-I.

Active exogenous antigen (protein and particle) uptake and degradation, a necessary upstream process for exogenous antigen presentation including cross-presentation, has been documented in LNSCs. In addition to protein and particle uptake by LECs *in vitro* (147, 148), fluorescently labeled OVA has been detected in LYVE-1^+^ LECs within minutes of intradermal injection *in vivo* (148). In a similar study, intracellular antigen degradation and processing was visualized by increased DQ-OVA fluorescence within 90 min of subcutaneous injection (17). A recent report suggested that LECs can retain antigen over extended time periods, with detectable OVA fluorescence in LECs even at 1–3 weeks after injection. However, the same report also described that DQ-OVA fluorescence, delivered with TLR agonists and anti-CD40, was no longer detectable in LNSC populations by flow cytometry a week after injection (97). This suggests that more than one pathway of OVA uptake or intracellular trafficking is active in LECs such that ingested antigens may be trafficked and processed differently when antigen reaches the cells together with inflammatory signals, such as TLR agonists and anti-CD40 (162–164).

Such feedback mechanisms where the composition of an ingested antigen influences antigen trafficking are known to exist on professional APCs. Scavenging receptors can bind to antigens associated with heat shock proteins and other chaperone proteins, with resulting cross-presentation of the antigen (165, 166). They can also interact with TLRs, and affect the immunological phenotypes of APCs, such as the polarization of macrophages (167). Engagement of one such receptor, the mannose receptor, has been shown to route the binding antigen to a cross-presentation pathway (168). Further facilitating MHC-I processing, subsequent ubiquitination of mannose receptor can lead to cytoplasmic escape of the mannose receptor bound antigen, enabling easier access to MHC-I loading machinery (169, 170). Although cross-presentation, involving these mechanisms, has been shown thus far in model cell lines and in DCs, it cannot be ignored that the majority of the scavenger receptor superfamily (167), other known cross-presentation receptors, and C-type lectins are abundantly expressed by skin draining and mesenteric LNSCs (Figure S1 in Supplementary Material). Together with their expression of other proteins important in cross-presentation and a number of TLRs (146) (Figure S1 in Supplementary Material), the existence of these scavenging pattern recognition receptors (PRRs) on LNSCs may play a role in the cross-presentation of exogenous antigens, thus shaping immunological outcomes.

While the importance of scavenging receptors on LNSC immunobiology is only beginning to be appreciated, their gene expression pattern may help explain the preferential accumulation of the intradermally administered OVA in LECs versus in other LNSCs (97, 148). This LNSC distribution is somewhat unexpected because OVA is a low molecular weight protein (45 kDa), and therefore FRC and BEC association would follow the expected drainage pattern (Figure 1). However, because OVA is mannosylated (171), this may preferentially drive internalization by mannose receptor CD206 (172), which amongst the LNSCs, is present only on LECs (Figure S1 in Supplementary Material). This phenomenon may also be mirrored in the uptake and persistence of Herpes Simplex Virus glycoprotein in LECs (97), as baculovirus production of antigens can result in N-glycans with high-mannose glycosylation patterns (173).

There are also a number of other major cross-presentation pathways that have yet to be investigated, and appear to play minor roles in LNSC. Although it has been shown that DCs efficiently cross-present immune complexes upon rapid internalization by Fcγ receptors (174–176), LNSCs do not seem to express high levels of activating nor inhibitory Fcγ receptors (Figure S1 in Supplementary Material). The one exception is FcRn, which is expressed across all LNSC. Better known for its IgG recycling and transport functions in neonatal (177, 178) and drug delivery contexts (179), FcRn binds to IgG Fc and albumin in acidic environments and regulates their homeostatic levels in the circulation (180). FcRn also binds antibody-coated antigens, which has implications in antigen recycling, antigen archiving, MHC-II presentation as well as MHC-I presentation (175, 181). Another example involves the complement receptors, which are constitutively active in professional APCs, but are largely absent in resting LNSCs. However, C1q receptor for phagocytosis (C1qrp) stands out as an exception (Figure S1 in Supplementary Material). In professional APCs, C1q has been suggested to favor cross-presentation as seen by priming of antigen-specific T cells, but the direct involvement of C1q receptor in this process was not demonstrated (182). Given that multiple C1q-binding partners are known (183, 184), the functional utility of C1q receptor on LNSCs remains to be clarified.

Whether any of these transcriptional findings of antigen scavenging receptor genes on resting state LNSCs have productive functions in antigen presentation is an open question. In addition, the efficacy of cross-presentation mainly studied in professional APCs depends on physical properties of the antigen (162, 185, 186). Furthermore, uptake mechanisms can determine the fate of the internalized antigen in one of the several trafficking pathways depending on the APC type and the concurrent inflammatory signals (163). These mechanisms are only beginning to become elucidated in LNSCs. Nevertheless, it is clear that more work needs to be done to understand the cross-presentation pathways in LNSCs.

#### Consequences of Direct MHC-I Presentation by LNSC

One of the most important hypotheses to describe the activation of CD8^+^ T cells involves a three-signal mechanism, which stipulates that naïve cells will need to (1) recognize their cognate antigen presented in the context of an MHC-I molecule, (2) receive a co-stimulatory signal through their co-stimulatory receptors, and (3) be primed by cytokines (187). The impact of the first signal, direct MHC-I presentation with LNSCs playing the role of APCs, has been extensively reviewed in the recent years (1, 24, 36, 155, 188–196). To summarize, peptide:MHC-I complexes on LNSC surfaces resulted in initial antigen-specific CD8^+^ T cell proliferation in all of the models used. Following the hypothesis, antigen presentation alone without the second signals – signaling through co-stimulatory receptors – will not lead to fully functional T cell activation. Notably, these second signals are lacking in LNSCs (Figure S3 in Supplementary Material), which have a negligible level of expression of co-stimulatory molecules compared to DCs. Furthermore, LNSCs do not upregulate the co-stimulatory molecules CD40, CD80, and CD86 following stimulation with inflammatory cues, for example, following TLR engagement (most studies have used TLR3 agonist polyI:C), or in presence of IFN-γ or TNF-α (18, 146–148, 197). While LECs upregulate the immunostimulatory molecules HVEM, CD48, and MHC-II under such conditions (197), they also concomitantly upregulate PD-L1, as do FRCs, and BECs under the same inflammatory conditions (146, 148, 197, 198). Such observations explain findings, describing the early generation apoptosis of antigen-specific CD8^+^ T cells following co-culture with antigen-loaded LEC *in vitro* (148, 197). Furthermore, LEC-educated CD8^+^ T cells express less IFN-γ or IL2 than DC-educated counterparts, and cannot be reactivated by the addition of IL2. *In vivo* LNSC antigen-presentation studies have also reported poor recovery of transferred antigen-specific T cells (135, 146, 152–155, 158), due to inhibitory LNSC-CD8^+^ T cell crosstalk via the PD-L1:PD-1 and MHC-II:LAG3 (135) signaling axes. Thus, with the exception of one report using a model of PTA expression (134), most studies have suggested that direct antigen presentation by LNSCs, specifically by LECs and FRCs, appears to promote a dysfunctionally activated phenotype in interacting CD8^+^ T cells, which can result in deletional tolerance.

### MHC-II presentation by LNSC

The hallmark of professional APCs, namely DCs, macrophages, and B cells, is the constitutive cell surface presence of MHC-II and their proven ability for antigen processing and presentation (133). Constitutive MHC-II expression is restricted to a small number of cells of the immune system. Nonetheless, as reviewed recently (199, 200), there are now many different cell types from both hematopoietic and non-hematopoietic origins that can indeed express MHC-II and interact with CD4^+^ T cells in the periphery.

#### MHC-II Expression by LNSCs

LNSCs constitute such non-professional APC cell types that express MHC-II. Unlike MHC-I molecules, however, MHC-II expression is highly regulated. The class II transactivator (CIITA) is the master regulator for the expression of MHC-II. This transcription factor forms a DNA-binding multiprotein complex that controls MHC-II expression in all MHC-II expressing cell types (201). CIITA expression itself is regulated by cell-specific promoters, of which three have been identified, namely pI, pIII, and pIV. Amongst them, the promoter pIV is essential for driving constitutive CIITA expression in thymic epithelial cells and for mediating IFN-γ-dependent MHC-II expression in cells of non-hematopoietic origin, such as endothelial cells, epithelial cells, fibroblasts, and astrocytes (202). Although MHC-II expression in human umbilical vascular and foreskin endothelial cells can be induced by lymphocyte adhesion in a CIITA-independent fashion (203–205), recent evidence demonstrates the major role of pIV-driven CIITA for MHC-II regulation in LNSCs.

The first observation of MHC-II expression on endothelial cells was reported more than 30 years ago (206). The capacity of human blood and lymphatic endothelia to express MHC-II was believed to be linked to cytokine stimulation via IFN-γ or TNF-α (207–209). For example, MHC-II expression on an immortalized murine FRC cell line also depended on IFN-γ (210). In recent studies, it was demonstrated that BECs, but not LECs from skin explants, constitutively express MHC-II, revealing functional differences prompted by environmental factors (211). Even more recently, steady-state MHC-II expression in LN LECs, BECs, and FRCs has been reported at both transcriptional and protein expression levels (8, 91, 134, 147). By using transgenic mouse models lacking the different CIITA promoters, steady-state levels of MHC-II molecules on the surface of LNSCs were demonstrated to be a combination of IFN-γ-inducible, pIV-driven CIITA basal activity, and acquired MHC-II complexes from DCs (198). *In vivo*, LNSCs from CIITA pIV^−/−^ mice and IFN-γ receptor-deficient mice exhibit an identical MHC-II profile that was above background levels, pointing to other contributions. This suggests that differences in MHC-II expression levels observed in published studies may arise from environmental factors, for example, from differences in animal facilities. In addition to endogenous pIV-mediated MHC-II expression, LNSCs are capable of acquiring MHC-II from DCs as demonstrated in irradiated MHC-II-deficient mice re-constituted with MHC-II-expressing bone marrow (BM) precursors. In these studies, radioresistant MHC-II-deficient LNSCs displayed significant levels of surface MHC-II molecules compared to LNSCs in mice re-constituted with MHC-II-deficient BM. This is supported *in vitro* by observations that following removal of hematopoietic cells, LEC and FRC cultures lack any expression of MHC-II unless co-cultured with DCs. *In vivo*, manipulation of DC numbers in LNs significantly affects levels of MHC-II expressed by LECs, BECs, and FRCs, demonstrating the existence of intercellular MHC-II transfer from DCs to LNSCs *in vivo*.

#### Antigen Transfer and LNSCs in Antigen-Specific CD4^+^ T Cell Responses

Antigen transfer can occur as a peptide exchange on cell surfaces as well as the transfer of peptide:MHC complexes through membrane exchange between cells.

In peptide exchange, peptide epitopes can bind directly on cell surface or early endosomal MHC molecules (212–216), where both MHC-I and II are receptive to binding lymph-borne peptides. This is particularly relevant as recent analysis of the human lymph peptidome showed a predominantly self-peptide repertoire, including products deriving from extracellular processing of proteins, rather than that of the traditional repertoire derived from intracellular MHC-II processing (43, 217–220). While these peptides have not been shown to actually bind to LNSCs, these cells are ideally localized to fully sample these low molecular weight peptides.

Membrane exchange between cells is not an uncommon occurrence in biology and immunology (221). In the LN, for example, blebs of CD169^+^ macrophages are acquired by innate-like T cells and NK cells under steady-state conditions (222). Peptide:MHC-I and MHC-II complexes have been shown to be transferred between DC and tumor cells (223) as well as between DCs (224). In another example, MHC-II has been demonstrated to be present on exosomes secreted by DCs (225, 226).

Between hematopoietic APCs and LNSCs, transfer of antigens and peptide:MHC-II complexes seems to play an important role in MHC-II restricted antigen presentation. Exosomal transfer appears to represent a major route of MHC-II transfer from professional APCs to LNSCs. While FDCs are not able to synthesize MHC-II themselves (227) nor phagocytose antigens (228), human tonsillar FDCs have been reported to retain exogenous MHC-II vesicles (229). By immunoelectron microscopy, they were thought to be exosomes due to their protein immunoreactivity, and were possibly peptide loaded. While the source of the exosomes was not clarified (B cells or DCs), such MHC-II peptide complexes on FDC cell surface can interact with CD4^+^ T cells. Exosomes were also implicated in the transfer of peptide:MHC-II complexes from DCs to LNSCs (198). The transfer of peptide:MHC-II complexes from DCs, but not from B cells or macrophages, maintained the steady-state levels of MHC-II on FRCs, LECs, and BECs (198). Consistent with known surface marker expression patterns on LNSCs, if the transfer of DC co-stimulatory molecules occurred, it did not result in detectable forms.

Antigen transfer between LECs and DCs is however, not restricted to one direction. Indeed, the transfer of transgenic LEC-specific PTA to hematopoietic cells has been described (135). Neither membrane-bound nor cytoplasmically expressed PTAs were presented by LECs to prime antigen-specific CD4^+^ T cell responses (135). This was attributed to the orders of magnitude lower expression of H2-M in LECs compared to professional APCs, which is required to free the MHC-II groove for peptide binding. Instead, the model PTA β-gal expressed by LECs was shown to be loaded onto MHC-II in hematopoietic cells (135). While the exchange mechanism is still open to examination, it was reported not to be dependent on recognition of apoptotic cells or DC phagocytosis. On the other hand, FRCs and BECs express the adaptor molecule H2-DM under steady-state and upon inflammation (8, 134) (Figure S2 in Supplementary Material). Despite this key difference, it remains unclear whether FRCs or BECs may act solely as PTA reservoirs or, in addition, can directly present antigen and impact CD4^+^ T cells. Supporting direct CD4^+^ T cell priming by FRCs, the aforementioned immortalized FRC cell line was shown to induce CD4^+^ T cell proliferation *in vitro* (210).

These complementary bidirectional observations highlight the close relationship and communication between professional APCs and LNSCs to enable MHC-II presentation by LNSCs.

As the nature and regulation of MHC-II expression in LNSC has only recently begun to be elucidated, the functional contributions of LNSC antigen presentation to CD4^+^ T cells remain unclear in part due to its complexity. Lack of measurable productive T cell responses has been one of the major difficulties preventing the clarification of the impact of antigen presentation by LNSCs on CD4^+^ T cell outcome. As for CD8^+^ T cell responses, the absence of co-stimulatory signals, such as CD80 or CD86 and the constitutive expression of PD-L1 by LNSCs (197, 198), preclude the possibility of functional effector CD4^+^ T cell priming. In other words, MHC-II expression is mandatory but not sufficient to elicit a measurable CD4^+^ T cell response. In this regard, it has been shown that human LN-derived LECs fail to induce allogeneic CD4^+^ T cell proliferation even after IFN-γ stimulation (147). In these particular *in vitro* settings, LECs were unable to induce proliferation of either naïve or memory CD4^+^ T cells. Likewise, DC-acquired peptide:MHC-II complexes presented by murine LNSCs to cognate CD4^+^ T cells not only failed to promote T cell proliferation but also, in the case of LECs, induced increased cell death in an antigen-dependent manner. Moreover, this process led to CD4^+^ T cell unresponsiveness to further restimulation with anti-CD3 and anti-CD28 antibodies (198). These challenges present a major obstacle in understanding the relative contributions of acquired peptide:MHC-II complexes and endogenous MHC-II molecules to CD4^+^ T cell responses *in vivo*.

A recent study described the role of LNSC MHC-II in maintaining antigen-specific regulatory T cells (Tregs) (134). An elegant model of LN transplantation was used, where MHC-II-deficient LN is transplanted into wild-type recipients (Table S1 in Supplementary Material). In this model, the LNSCs remain MHC-II deficient, while MHC-II-sufficient hematopoietic cells repopulate the transplanted LN. This local absence of MHC-II expression in LNSCs was directly correlated with a reduction in Treg numbers. Importantly, only local Treg numbers were affected, and neither wild-type transplant control nor endogenous distal LNs exhibited the same effects.

However, the specific contribution of each LNSC subset remains to be determined. Indeed, with regards to LEC-restricted features, such as high levels of PD-L1 expression (156) as well as non-overlapping PTA expression in LECs, BECs, and FRCs (146, 152–155), it is likely that each LNSC subtype is capable of impacting CD4^+^ T cell outcomes, including Treg development and differentiation. Moreover, antigen-processing and MHC-II-mediated antigen presentation by LNSCs still needs to be carefully dissected, especially in a context where antigen exchange between neighboring cells has been demonstrated.

In summary, LNSCs possess important roles under physiological conditions, and the function of these cells as APCs also appears to be crucial during ongoing immune responses and inflammatory conditions. The fact that the main stromal cell subsets indeed upregulate MHC-II expression in a IFN-γ-dependent manner implies a greater ability of these cells to influence CD4^+^ T cell responses in inflamed LNs. In fact, LNSCs have been suggested to constrain CD4^+^ T cell expansion after viral challenge (230). The nature of such effects, however, has not been deeply investigated and the conclusions remain to be clarified.

### Shared responsibilities

It is striking that many of the mechanisms and functional consequences of antigen presentation are shared across the LNSC subsets, with the exception of FDCs. The described MHC-I and II presentation are much the same between FRCs and LECs, and although less studied, some aspects are also similar in BECs. These three LNSC subtypes are IFN-γ responsive through expression of IFN-γ receptor (8). In FRCs and LECs, PD-L1 expression and lack of co-stimulation seem to be important in their education of CD8^+^ T cells. Furthermore, key chemokine and cytokine expression supporting T cell education is distributed amongst FRCs, LECs, and BECs, for example, CCL21 expression for attracting CCR7^+^ DC and T cells (FRCs, LECs, and BECs) (231), and IL7 for supporting naive T cells (FRCs and LECs) (5, 232–234), and IL15 for the development and homeostasis for naïve and memory T cells and other cells (FRCs, BECs, and DN) (235) in the LN. As the understanding of LNSC antigen presentation deepens, the shared characteristics between LNSCs must be reconstructed with the LN microarchitecture and antigen flow in mind.

### Challenges in studying LNSC antigen presentation

Lack of suitable *in vivo* models presents a major difficulty in demonstrating the relative importance of antigen presentation by LNSC subsets. Directly studying the role of antigen presentation in each LNSC subset, including specific targeting, capture, and processing of exogenous antigens is not easily accomplished *in vivo* (Table S1 in Supplementary Material). While depletion of hematopoietic cell subtypes is routine and well-established, ablation of any of the stromal subsets results in a major disruption of the LN microenvironment and organization, and any immunological effects of LNSC antigen presentation become difficult to interpret due to experimental artifacts. In this regard, while care must be taken to exclude the contribution of radioresistant APCs (134, 236), BM chimeras and LN transplants represent less drastic alternatives that have begun to enable the deconvolution of hematopoietic from stromal contributions to antigen presentation. Concerning MHC-II, the CIITA pIV^−/−^ mice can be used as a model for MHC-II abrogation in all non-hematopoietic cells, including LNSCs (237, 238).

Supposing the antigen presentation can be limited in one LNSC subtype, another challenge in studying antigen presentation by LNSCs is the reliance on T cell proliferation as a practical read-out. In addition to the balance favoring coinhibitory to co-stimulatory molecules, LNSCs can suppress T cell activation in a contact-dependent manner (18–20). As T cell proliferation integrates several outcomes deriving from LNSC and T cell contact, not limited to antigen peptide:MHC and T cell receptor recognition, other reporting systems of direct antigen presentation need to be developed.

What have been rewarding are the models of LNSC subset-restricted PTA expression (Table S1 in Supplementary Material). Direct presentation of PTAs by LNSCs has been established largely by using two animal disease models: inflammatory bowel disease (146, 152, 239) and autoimmune vitiligo (153, 154, 156, 197, 240), paired with T cells harvested from respective antigen-specific TCR transgenic mice.

Useful in addressing the challenge of LNSC-restricted antigen presentation and PTA expression, a number of mouse models are available in which antigen-presentation machinery can be knocked out or altered in a stromal cell-specific manner. This selectivity can be accomplished using the Cre-lox recombination system, in which target genes marked by loxP recombination sites are excised by a Cre recombinase enzyme expressed under a cell-specific promoter (241). Especially in conditionally inducible form, these transgenic mouse systems present a useful means to study the immunological roles restricted to each of the stromal subsets. Examples of this, some of which have already been mentioned above, include the CCL19-Cre system for FRC-specific knockouts (242), and the LYVE-1-Cre and inducible Prox1-CreER^T2^ systems for LEC-targeted knockouts (27, 243, 244). As with any genetic model, there are caveats. For example, using LYVE-1-Cre, deletion of floxed genes has been observed in some hematopoietic cells (27). While sparing the major APCs of the LNs (135), PROX-1, which is essential for lymphatic development and maintenance (245), have been shown to be expressed by several other cell types in multiple organs (246). Depending on the experimental goals and objectives of a study, both Cre lines may need to be used as complementary models to evaluate the immunological outcomes of LEC-specific PTA expression (135). Podoplanin-Cre has been described to target both LECs and FRCs, and has contributed to the discovery of a new stromal subset found in spleen (247). For BECs, Tie2-Cre (248) and Flk1-Cre (249) have been described (244). It must be noted that both Tie2 and Flk1 are expressed in cells of hematopoietic origin (250, 251), requiring BM chimeras to limit the effect of Cre recombination to stromal cells. FDCs have been targeted by using Cd21-Cre (252), and their developmental origins have been explored by crossing Cd21-Cre with the Ubow mice in fate-mapping studies (10). Again, it is notable that Cd21-Cre-mediated deletion was also observed in other tissues, including the forebrain (253).

In a few cases, these LNSC subset-targeting Cre mice have been crossed with available mouse models carrying floxed MHC-I and II genes. In fact, MHC-II flox has been successfully used with Tie2-Cre to eliminate MHC-II expression in both the hematopoietic and endothelial lineages, while MHC-II expression remains unaffected in the thymic epithelial cells (250). The naïve CD4^+^ T cell numbers were comparable with Cre-negative littermates, while numbers of Tregs and antigen-experienced cells were significantly decreased in the knockouts. As for generating MHC-I conditional knockouts to be used with Cre systems, mice with β2M floxed allele can be generated in two steps (254).

Aside from genetically modified animal models, other mechanisms of specifically targeting molecules responsible for antigen uptake and processing in LNSC subsets have not been actively explored (255). Once targeting can be achieved, relevant methods exist to modulate MHC-I antigen presentation, for example, to inhibit MHC-I loading of peptides using viral inhibitors (128).

Another limitation of drawing any general conclusions for antigen presentation by LNSC derives from the fact that often, LNSC are defined as bulk populations, based on CD45, gp38 and CD31 expression. Recent papers have delved deeper into nuances of the LNSC subtypes beyond those defined by these surface markers. The findings demonstrate the need to further subdivide LNSC based on their anatomical location, molecular phenotype, and functional differences (2, 4, 38). For instance, the grossly defined FRC population contains marginal reticular cells (256), and the BEC population can be functionally distinguished as being composed of HEVs and capillary endothelial cells (91). Finally, aside from the recently identified integrin-α7-expressing pericytes (8), and gp38^−^ FDC (2, 11), the DN fraction (gp38^−^ CD31^−^) has yet to be fully defined. Thus far, no animal models address these subdivisions. More transgenic mouse models precisely engineered (257, 258) to study antigen presentation by LNSC subsets may become available in the near future.

Alternatively, *in vitro* models may be used to investigate antigen presentation in LNSC subtypes. These models present technical difficulties of their own, especially when using sorted primary LNSCs. LNSC subtypes are not only defined by surface markers and cytokine production profiles but also by their biochemical and biophysical communication with other cells. This interdependence can be indirectly appreciated from transcriptome studies comparing freshly isolated versus cultured human LECs and BECs (211), which demonstrated that some key characteristic gene expression signatures of LECs and BECs were lost following *in vitro* culture. Another study compared freshly isolated human LN FRCs to LN FRCs propagated over 60 days in culture, and arrived at similar conclusions (259).

Such loss of phenotype may be potentially averted in several ways. For instance, physiological complexity can be introduced to existing *in vitro* LNSC co-culture systems by engineering an environment that recapitulates physiologically inferred mechanical tension and fluid flow (260). For primary FRCs, CCL21 expression, which is otherwise lost following *in vitro* culture, was rescued by growing the cells under conditions mimicking interstitial flow rates on a 3D composite matrix capable of mechanically supporting cell tension (261). Moreover, FRC co-culture with lymphocytes in a semi-3D nylon mesh promoted the generation of a robust reticular network structure (15). This further highlights that LNSC differentiation states and likely antigen-presentation properties are interdependent on biophysical and biochemical cues between the neighboring cells and the microenvironment.

Inescapably, the LN architecture must be reconstructed to grasp the relevant cell-cell interdependencies in the context of their LN locations. Established slice cultures (115), development of organoid cultures and complex *in vitro* systems (262) improves on conventional 2D co-culture systems to interrogate LNSC antigen presentation and immune cell education. Furthermore, dissecting the microanatomical differences of the LNSC subsets using techniques, such as histo-cytometry (23, 263), which yields multiparameter cell marker analysis coupled with anatomical location, may prove to be informative in understanding the role of LNSC antigen presentation within the complex coordination of antigen, DC and T cell interactions (264).

Nevertheless, *in vivo* models will remain a cornerstone in understanding LNSC antigen presentation as the biological complexity remains irreproducible *in vitro*. This includes the dynamics of the cellularity of the LNSCs, where each LNSC population differentially expands and contracts during an immune response upon viral infection and vaccination (94, 230). Two-photon intravital microscopy has greatly contributed to our understanding of the kinetics of antigen–cell and cell–cell interactions in the initiation of immune responses in the LN (265). These studies make use of genetically engineered mouse models, which express fluorescent proteins in specific stromal cells subsets: for LECs (246, 266–269), vascular endothelial cells (270, 271), and for LECs and BECs simultaneously (78, 272) [for review, see Ref. (244)]. So far, these mice have generally been used for real-time observations in organs other than the LN. However, the same mouse models may greatly contribute to studying the dynamic interactions of antigens, DCs and T cells with stromal cells (273, 274). By capturing early T cell interactions with antigen-presenting LNSCs, the issues of using T cell proliferation as a read-out may be avoided.

Much remains to be explored to understand the role of LNSCs as APCs in both pathological and physiological processes, such as wound healing, cancer, transplantation, and autoimmunity. Last but not least, most of the observations of LNSC antigen presentation have been made in mice, and need to be addressed in human LNs and diseases. In summary, phenotypic differences inherent to various LNSC subtypes, their anatomic locations, and their interactions with surrounding hematopoietic and stromal cells collectively determine their ability to coordinate antigen presentation and T cell education. Grasping the contributions of individual LNSC subtypes within this complex system remains elusive due to significant technical hurdles, thus emerging tools, models, and technologies are likely to be crucial to the elucidation of key mechanisms.

## Relative Contributions of LNSC Antigen Presentation on Immunological Outcomes

While there is accumulating evidence that LNSCs modulate peripheral tolerance and the initiation and resolution of immune responses through both direct and indirect mechanisms, the relative importance of LNSC versus DC antigen presentation has not been integrated into a larger model. Several mechanisms of direct interactions between LNSCs and APCs have been described, including active antigen transfer and direct sampling of antigens on LNSCs by APCs. Depending on the model system, hematopoietic APCs are highly important in LNSC-harbored antigen. For example, in BM chimera studies, there was no antigen-specific T cell proliferation if LECs are, but the hematopoietic APCs are not capable of presenting antigen (135). It is certain that DCs are the dominant actors in antigen presentation and tolerance induction against peripheral self-antigens (275). In addition to CD11c^+^ DCs, extrathymic Aire-expressing cells (eTACs) also appear to play an important role, namely in peripheral tolerance. eTACs are a radioresistant CD45^lo^ CD11c^lo^ CD11b^lo^ MHC-II^hi^ PTA-expressing APC of BM origin, which reside in the inter-follicular region in the LNs (276). eTACs share many features with antigen-presenting LNSCs, such as similarly low expression of CD80 and CD86, and high expression of PD-L1. This extends to their ability to initiate CD8^+^ T cell deletion (276). They further deactivate CD4^+^ T cell responses independently of the enriched antigen-specific Treg population (277).

Nevertheless, it is clear that the tipping point to initiate an immune response or to tolerate an antigen is dependent on a fine balance between antigen presentation by LNSCs or by APCs of hematopoietic origin.

### Initiation and maintenance of the immune response

The importance of LNSC antigen presentation can be appreciated in studies of infections in BM chimeric mice. It was shown that during pathogen infection, non-hematopoietic cells have a noticeable role in initiating antigen-specific CD4^+^ T cell responses (230) and CD8^+^ T cell proliferation (278, 279). When LNSCs could not present antigen, the overall clonal expansion was diminished in several viral infection models, suggesting a role of LNSC antigen presentation for early clonal CD4^+^ and CD8^+^ T cell expansion. This initial clonal expansion in the CD4^+^ T cell populations were 10× less than when the antigen is sterile, i.e., non-replicating (230), which points to the importance of the amount of available antigen. On the other hand, to sustain the CD8^+^ T cell expansion, hematopoietic APCs were reported to be necessary (279).

In these settings, even when focusing on the levels of LNSC antigen retention and presentation alone, the interpretation is difficult and does not necessarily correlate with the T cell proliferation response. When compared side by side, the magnitude of LNSC-primed CD8^+^ T cell responses differs with the infectious agent (279). The least T cell proliferation upon LNSC antigen presentation was seen in *Listeria monocytogenes* infections (279), which is thought to mainly target macrophages, DCs, and hepatocytes (280) and not be harbored in LECs (97). However, the greatest effect of LNSC antigen presentation on T cell proliferation was shown for the LCMV Armstrong strain, which unlike clone 13 of the same virus, does not extensively infect FRCs (95). Rather, like in an infection with strain WE, the Armstrong infection results in FRC network destruction and decreased long-term antigen presence in the LN (95, 96). Intermediate contributions of LNSC antigen presentation were supported by T cell responses to vaccinia virus, which is archived in LECs (97), and in VSV infections, both of which infect sessile cells just below the SCS (281). Thus, systematic analysis must be conducted to understand how LNSC antigen retention and presentation aid the initiation of the antigen-specific T cell response.

### Distinct T cell differentiation states and immunological protection

In light of existing data, an attractive hypothesis is that LNSC antigen presentation contributes to the generation of diverse T cell phenotypes in the face of antigen-specific challenge. This hypothesis is inspired by observations that CD8^+^ T cells primed by cross-presenting LSECs, which were previously thought to be tolerized (282), then were shown to be reactivated under viral challenge (283). LSECs occupy a large surface area exposed to blood that carries external food and commensal bacterial antigens and are known to cross-present exogenous antigens (130, 160, 161). Similar to MHC-I restricted antigen presentation by LNSCs, antigen cross-presentation by LSECs resulted in tolerized CD8^+^ T cells, where LSEC PD-L1 expression was important (284, 285). The surviving LSEC-educated T cells had an antigen-experienced central memory-like phenotype in secondary lymphoid organs (283). Furthermore, LSEC-educated T cells could be reactivated *in vitro* and *in vivo* in an antigen-specific manner in the presence of CD28 and IL12, and they could participate in an antigen-specific viral challenge with recombinant adenovirus (283).

Given the similarities between LSECs and LNSCs, the observation that LSEC-educated T cells are not terminally tolerized, but are primed for reactivation, leads to the question of whether or not LNSC-educated T cells behave comparably. Earlier, we described how LNSCs appear to induce a dysfunctionally activated phenotype in interacting T cells, through their ability to present antigens but not the co-stimulatory signals required to induce T cell activation. Like LSEC-educated CD8^+^ T cells, LNSC-educated and tolerized CD8^+^ T cells in the iFABP-tOVA PTA model were reactivated when challenged with intravenously injected VSV-OVA (286) (Table S1 in Supplementary Material). The iFABP-tOVA mouse line with lower PTA expression levels resolved the infection and retained a cognate CD8^+^ T cell population after a VSV-OVA challenge, while the line with higher antigen expression levels suffered death by T cell mediated destruction of the PTA-expressing intestinal epithelial cells (286). These T cells were capable of target lysis, yet unable to secrete IFN-γ and TNF-α, and resided in inter-epithelial layers (286). For lack of an existing phenotypic category, the authors called this phenotype of the LNSC-primed T cell, split-anergy.

## Concluding Remarks

The immunological roles of LNSCs cannot be dissociated from the structural microarchitecture of the LN, albeit in a much more nuanced manner than previously appreciated. LNSCs can act at different levels to promote but also regulate antigen presentation both directly and indirectly by interacting with APCs and T cells. Moreover, the interactions and relationships between the different stromal cells together with DCs (11, 112, 287) are crucial in framing the immunological roles of LNSCs, which result in the physical and biochemical modulation of the LN microenvironment.

The immune system relies heavily on suppression under resting conditions and during resolution of immune responses. It is not surprising, then, that more layered and likely redundant cellular actors and intercellular interactions are involved in suppression of undesirable immune activation. The LNSC-educated T cells find a place in the recently articulated adaptation model of immunity that goes beyond the self/non-self recognition and danger theory to explain tolerance during an ongoing immune response: (1) recognition of self/non-self/mimic of self by T cells, (2) activation of the immune response, and (3) efficacy of the immune response (288). Within this framework, the efficacy of the immune response is programed by the expression and engagement of the sets of adaptation receptor–ligand pairs on the T cells and their interacting APCs/LNSCs (for example, PD-1/PD-L1, other co-stimulatory and coinhibitory molecules, adhesion molecules, and pattern recognition molecules) (288). At least in mouse models, LNSCs fulfill the first two requirements: antigen presentation to T cells, and the initiation of antigen-specific T cell responses. Furthermore, LNSCs are capable of interacting with and signaling through distinct sets of adaptation receptors on T cells from those on hematopoietic APCs (147). Interdependence of co-stimulatory and coinhibitory pathways generates another layer of complexity (197).

Antigen presentation in the LN takes place in a wide spectrum of differently timed and equipped antigen-presenting LNSCs and hematopoietic APCs, not to mention that the encounter with lymphocytes is highly influenced by the inflammation responsive LN microarchitecture. This results in ever-expanding and plastic phenotypes of antigen-experienced T cells. As more becomes known about the roles of LNSCs in antigen-specific responses, their contributions to adaptive immune responses must be considered especially when applying this knowledge to the engineering of vaccines and immunotherapies.

## Author Contributions

SH has developed the concept, wrote the manuscript, prepared the figure, supplemental figures and the table, and critically read, revised, and approved the manuscript. JD has co-developed the concept, co-wrote the manuscript and the supplemental table, critically read, revised, and approved the manuscript.

## Conflict of Interest Statement

The authors declare that the research was conducted in the absence of any commercial or financial relationships that could be construed as a potential conflict of interest.

## Supplementary Material

The Supplementary Material for this article can be found online at http://journal.frontiersin.org/article/10.3389/fimmu.2015.00446

## Abbreviations

APC(s), antigen-presenting cell(s); BEC(s), blood endothelial cell(s); BM, bone marrow; CIITA, class II transactivator; DC(s), dendritic cell(s); DN, double negative; DT, diptheria toxin; eTAC(s), extrathymic Aire-expressing cell(s); FDC(s), follicular dendritic cell(s); FRC(s), fibroblastic reticular cell(s); gp38, podoplanin; HEV(s), high endothelial venule(s); IFN-γ, interferon-γ; LEC(s), lymphatic endothelial cell(s); LN(s), lymph node(s); LNSC(s), lymph node stromal cell(s); LSEC(s), liver sinusoidal endothelial cell(s); MHC(s), major histocompatibility complex(es); OVA, chicken egg ovalbumin; PLVAP, plasmalemma vesicle-associated protein; PTA(s), peripheral tissue antigen(s); SCS(s), subcapsular sinus(es); TAP, transporter associated with antigen processing; TLR(s), toll-like receptor(s); TNF-α, tumor necrosis factor-α; Treg(s), regulatory T cell(s); VEGF, vascular endothelial growth factor; VSV, vesicular stomatitis virus.
