# Supplementary material for: Modes of Antigen Presentation by Lymph Node Stromal Cells and Their Immunological Implications
Source: Front Immunol. 2015 Sep 8;6:446. doi: 10.3389/fimmu.2015.00446 (PMC4561840; doi:10.3389/fimmu.2015.00446)
Supplement: Supplementary file 1 [file Table_1.DOCX]

**Supplemental Table 1: Mouse models used to study lymph node stromal cell antigen presentation**

The table summarizes the main animal models that have shed light on the immunological roles of lymph node stromal cell (LNSC) antigen presentation. Abbreviations are as follows: BEC = blood endothelial cell, BM = bone marrow, DT = diphtheria toxin, FDC = follicular dendritic cell, FRC = fibroblastic reticular cell, HA = hemagluttinin, LEC = lymphatic endothelial cell, LN = lymph node, LNSCs = lymph node stromal cells, OVA = chicken egg ovalbumin, PTA = peripheral tissue antigen

|  | **Mouse Strain** | **Model** | **CD4, CD8** | **Features** | **Reference** |
| --- | --- | --- | --- | --- | --- |
| PTA Expression (Collier et al., 2008) | | | | | |
|  | iFABP-tOVA | Truncated cytoplasmic OVA driven by the intestinal fatty acid binding protein for  expression in jejunum, ilium  Adoptive transfer of antigen specific TCR transgenic OT-I (CD8+ ) and OT-II (CD4+) | CD8  Potentially CD4 | - Used to establish direct presentation by LNSCs - Proliferation and deletion of adoptively transferred CD8+ T cells - Direct presentation by LNSCs and cross-presentation by CD8a+ DCs | (Lee et al., 2007;Gregg et al., 2010) |
|  | MHC-I: AAD x FH | Tyrosinase, endogenously expressed in murine melanocytes, presented in a chimeric MHC-I (peptide-binding human HLA-A*0201 and CD8-binding H-2D^d^)  Cross with FH mice, transgenic for TCR specific for MHC-I peptide Tyr _369-377_ | CD8 | - PTA Tyrosinase only expressed in LECs in the LN - Used to establish LECs can directly present PTAs for antigen specific CD8+ T cell deletion - Deletion not dependent on central tolerance, Langerhans cells, macrophages, DCs | (Nichols et al., 2007;Tewalt et al., 2012) |
|  | GFAP-HA | Influenza hemagluttinin driven by the glial fibrillary acidic protein promoter for expression in enteric glial cells  Adoptive transfer of HA-specific CD8+ TCR transgenic mouse, CL4 | CD8 | - Direct presentation by LNSCs and cross-presentation by CD8a+ DCs of the mLN - LNSCs were sufficient for deletion | (Magnusson et al., 2008) |
|  | K14-mOVA | OVA driven by the human kerain 14 promoter with expression in the skin, thymus |  | - PTA OVA is expressed in FRCs, LECs and BECs in the LN - Derived cell lines *in vitro* supports FoxP3+ CD4+ T cells - Used in LN transplant studies summarized below | (Baptista et al., 2014) |
| **Stroma specific model antigen expression (not restricted to the LN)** | | | | | |
| FDCs | RCd21^cre^ mDEL^loxp^ | Membrane bound Duck egg lysozyme (DEL) conditionally expressed by breeding with CD21^cre^. The antigen will be expressed on mature B cells and FDCs.  The mice receive bone marrow transplant to eliminate the DEL expression on B cells, with MD4 BM | B cells | - HEL-binding B cells do not transition to T2 stage - HEL-binding B cells cannot prime CD4+ T cells, and are more prone to apoptosis at the B-T border   Similar model shows a permissive role of FDC restricted PrP^c^ expression on prion infection in the spleen | (Yau et al., 2013)  (McCulloch et al., 2011) |
| FRCs and LECs | Pdpn-Cre x Rosa26^stop-LacZ^ | β-galactosidase is expressed only in gp38+ cells  Adoptive transfer of LacZ specific CD4+ T cells Bg2, CD8+ T cells Bg1 | CD4, CD8 | - Bg1 CD8+ T cells needs Bg2 CD4+ T cells to proliferate - Bg2 CD4+ T cells transferred alone proliferates | (Onder et al., 2011) |
| LECs | Prox-1 CreER^T2^ x  Lyve-1-Cre x  Rosa26^stop-HA^  Rosa26^stop-LacZ^ | β-galactosidase or HA is expressed only in either Prox-1+ or Lyve-1+ cells  Adoptive transfer of antigen specific CD4+ (Bg2, TS1) or CD8+ (Bg1, Clone 4) T cells | CD4, CD8 | - LEC present b-gal and hemagluttinin (HA) epitope on MHC-I to CD8+ T cells, but neither epitopes are presented on CD4+ T cells - When antigen is transferred to DCs, results in CD4 T cell anergy | (Rouhani et al., 2015) |
| BECs | Tie2-LacZ | β-galactosidase is expressed under the Tie2 promoter  Adoptive transfer of LacZ specific CD8+ T cells Bg1 | CD8  Possibility of CD4 | - No activation nor tolerization observed upon adoptive transfer, also in organ transplant settings - CD11c+ DCs cross-presented BEC expressed antigen for CD8+ T cell activation - LacZ transcripts also found in hematopoietic stem cells | (Bolinger et al., 2008) |
| **LN transplants** | | | | | |
|  |  | Balb/c WT  Adoptive transfer of OVA specific CD4+ T cells DO11.10  Similarly in C57Bl/6 with OT-II CD4+ T cells | CD4 | - Useful for demonstration of local antigen drainage and microenvironment - After transplantation from peripheral or mesenteric LN into the gut mesentery, the donor LNSCs remain, however, by week 8, the hematopoietic cells are of recipient origin. - Mesenteric and celiac LN transplant to the popliteal fossa, the hematopoietic cell exchange occurs in 5 weeks - Gut-draining LN support de novo Treg induction, which is dependent on CD11c+ DCs | (Ahrendt et al., 2008;Hammerschmidt et al., 2008)  (Cording et al., 2014) |
| FRCs  LECs  BECs |  | C57Bl/6.129S2-H2^delAb1-Ea^/J (MHC-II KO), transplanted into WT mice  Or K14-mOVA LN transplanted into WT mice  With adoptive transfer of OT-II CD4+ T cells |  | - Selective lack of MHC-II on LNSCs in the transplanted popliteal LN results in transplant rejection after 8 weeks - CD4+ T cell depletion increases CD8+ T cell activation (CD62L- CD44+) systemically - LNSC expression of mOVA supports FoxP3+ OTII CD4+ T cells | (Baptista et al., 2014) |
| **Stromal subset ablation (not restricted to the LN)** | | | | | |
| FDCs |  | FDC ablation by anti-LTβR-Ig or anti-LTβ |  | - Decoy receptor for FDC survival factor lymphotoxin LTα1β2 - Induces rapid loss of FDC networks in spleen and LNs (24h), and loss of immune complex trapping in the follicles | (Mackay and Browning, 1998) |
| FDCs | CD21cre x DTR^flox^ | Selective diphtheria toxin (DT) -mediated ablation of CD21^+^ FDCs  The mice receive bone marrow transplant to allow for toxin-resistant CD21^+^B cells  (The mice also receive DT-neutralizing polyclonal antibodies to maintain low circulating corticosteroid levels) |  | - Ablation of FDCs in spleen and LNs (mice live up to 2 days) - The follicular stromal cell network remains largely intact in the spleen but not in the LN - Primary follicles become disorganized, mixing with DCs and T cells - Reduced total tissue CXCL13 transcript levels suggesting that FDCs are a source of this chemokine - Ablation of GC FDCs leads to disappearance of GCs and abrogates GC responses - FDCs are required for GC B cell clustering in the spleen and pLN but may be redundant in mLNs | (Wang et al., 2011) |
| FRCs | Ccl19cre x DTR^flox^ | Selective DT-mediated ablation of CCL19^+^ FRCs | CD4, CD8 | - Selective deletion of FRCs in the LN with no effect in cellularity of other LNSC populations. Deletion of FRCs and DN cells in splenic white pulp - Significant reduction in the size, weight and cellularity of the LNs but maintained integrity and permeability of the conduit network - Impaired adaptive CD4+ T cell responses: Reduced priming of antigen-specific T cells, activation and proliferation in the LN - Impaired virus-specific T cell responses: Reduced numbers of antigen-specific CD8+ T cells and defective interferon-γ production in the LN - Reduced B cell numbers in the LN and spleen. Disorganized B cell zone and LN cortex with mixing of B and T lymphocytes in the LN and altered white pulp architecture in the spleen - Impaired humoral responses: significant reduction in the production of influenza-specific antibodies in the LN and decrease in IgM production in the marginal zone of the spleen upon immunization - Highlights a new role of FRCs in the B cell follicles of the LN: regulation of B cell viability via production of the cytokine BAFF | (Cremasco et al., 2014) |
| FRCs | DM2 | Selective DT-mediated ablation of FAP+ FRCs |  | - Selective deletion of FRCs in the LN with no effect in cellularity of other LNSC populations for at least 10 days after DT treatment. - Significant reduction in the cellularity of the LNs, spleens are relatively unaffected (splenic FRCs not FAP+) - Deficient homing of naïve CD8+ T cells - Impaired humoral responses: decreased GC, TfH plasma cells, the affinity maturation is preserved - Decreased number of virus-specific CD8+ T cells - Depletion during an influenza infection only results in loss of naïve T cells, not activated T and B cells. | (Denton et al., 2014) |
| LECs | Lyve-1cre x DTR ^flox^ | Selective DT -mediated ablation of Lyve-1^+^ LECs |  | - Deletion of Lyve-1+ lacteals in the small intestine and Lyve-1+ lymphatic vessels in all LNs (including the inguinal, mesenteric, axillary, and cervical LN) but not in the skin, tracheal mucosa, and diaphragm after systemic administration of DT - Loss of lymphatic vessels in the LN mice causes aberrant localization of dendritic cells and sinusoidal macrophages but the distribution of T cells and B is not notably altered at 24 hours after DT administration - These mice die within 60h after DT administration without notable peripheral edema and erythema - Deletion of Lyve-1+ lacteals causes distortion of blood capillaries and whole architecture of the villi, and leads to severe inflammation of the proximal part of the intestine and sepsis - This model can be useful for local ablation of lymphatics in the ear: lymphatic vessels of the ear skin and draining cervical lymph node are ablated and lymph drainage is impaired after intradermal administration of DT | (Jang et al., 2013) |
| **Stromal Cell restricted MHC presentation (Bone marrow BM chimeras)** | | | | | |
| BECs | B6.C-H2^bm1^ BM transferred to Tie2-H-2K^b^ host | Only BECs have the H-2Kb that recognize the CD8+ epitope of OVA  Adoptive transfer of OT-I CD8+ T cells | CD8 | - Orally administered OVA presented on liver sinusoidal endothelial cells (also mesenteric LN) resulted in CD8+ T cell tolerance | (Limmer et al., 2005) |
| FRCs  LECs  BECs | B6 BM transferred to B6.C-H2-Ab1^bm12^ (bm12) mice | Only WT hematopoietic cells can present OVA due to mutation in the H2-Ab gene  Adoptive transfer of OT-II CD4+ T cells | CD4 | - Upon OVA/CFA immunization, OT-II CD4+ T cells expand, but contract slowly when LNSCs cannot present antigen, as evidenced by more Tfh cells remaining at later time points | (Abe et al., 2014) |
| FRCs  LECs  BECs | CD11cDOG BM transferred to CIITA^-/-^ host mice | LNSCs do not express endogenous MHC-II while hematopoietic cells do  OVA_140-386_ is expressed in CD11c+ cells | CD4 | - Any empty or peptide-MHC-II complexes are derived from the hematopoietic cells, and OVA CD4+ and CD8+ epitopes derive from CD11c+ DCs - LNSCs acquire OVA_323-339_–MHC-II complexes from DCs *in vivo* and induce an antigen-specific inhibition of OT-II survival and proliferation (*ex vivo*) | (Dubrot et al., 2014) |
| FRCs  LECs  BECs | β2m^-/-^ (B6.129P2-*B2m^tm1Un/J^* ) BM transferred to B6 host mice | LNSCs express MHC-I and can present antigen while hematopoietic cells cannot  Inoculation of B16F10 OVA-VEGFc expressing tumor cell line followed by adoptive transfer of OT-I CD8+ T cells | CD8 | - MHC-I on LNSCs support the proliferation of OT-I cells in the draining LNs of OVA-expressing tumors | (Lund et al., 2012) |
| LECs  (FRCs  BECs) | BM8 (B6.C-H-2K^bm8^) BM transferred to B6 host mice | LNSCs express MHC-I and can present antigen while hematopoietic cells cannot  adoptive transfer of OT-I CD8+ T cells |  | - Hematopoietic cells are required to present persistent antigen harbored in LECs | (Tamburini et al., 2014) |

**References**

Abe, J., Shichino, S., Ueha, S., Hashimoto, S., Tomura, M., Inagaki, Y., Stein, J.V., and Matsushima, K. (2014). Lymph node stromal cells negatively regulate antigen-specific CD4+ T cell responses. *J Immunol* 193**,** 1636-1644. doi: 10.4049/jimmunol.1302946.

Ahrendt, M., Hammerschmidt, S.I., Pabst, O., Pabst, R., and Bode, U. (2008). Stromal cells confer lymph node-specific properties by shaping a unique microenvironment influencing local immune responses. *J Immunol* 181**,** 1898-1907.

Baptista, A.P., Roozendaal, R., Reijmers, R.M., Koning, J.J., Unger, W.W., Greuter, M., Keuning, E.D., Molenaar, R., Goverse, G., Sneeboer, M.M., Den Haan, J.M., Boes, M., and Mebius, R.E. (2014). Lymph node stromal cells constrain immunity via MHC class II self-antigen presentation. *Elife* 3. doi: 10.7554/eLife.04433.

Bolinger, B., Krebs, P., Tian, Y., Engeler, D., Scandella, E., Miller, S., Palmer, D.C., Restifo, N.P., Clavien, P.A., and Ludewig, B. (2008). Immunologic ignorance of vascular endothelial cells expressing minor histocompatibility antigen. *Blood* 111**,** 4588-4595. doi: 10.1182/blood-2007-09-114769.

Collier, A.Y., Lee, J.W., and Turley, S.J. (2008). Self-encounters of the third kind: lymph node stroma promotes tolerance to peripheral tissue antigens. *Mucosal Immunol* 1**,** 248-251. doi: 10.1038/mi.2008.19.

Cording, S., Wahl, B., Kulkarni, D., Chopra, H., Pezoldt, J., Buettner, M., Dummer, A., Hadis, U., Heimesaat, M., Bereswill, S., Falk, C., Bode, U., Hamann, A., Fleissner, D., Huehn, J., and Pabst, O. (2014). The intestinal micro-environment imprints stromal cells to promote efficient Treg induction in gut-draining lymph nodes. *Mucosal Immunol* 7**,** 359-368. doi: 10.1038/mi.2013.54.

Cremasco, V., Woodruff, M.C., Onder, L., Cupovic, J., Nieves-Bonilla, J.M., Schildberg, F.A., Chang, J., Cremasco, F., Harvey, C.J., Wucherpfennig, K., Ludewig, B., Carroll, M.C., and Turley, S.J. (2014). B cell homeostasis and follicle confines are governed by fibroblastic reticular cells. *Nat Immunol* 15**,** 973-981. 10.1038/ni.2965.

Denton, A.E., Roberts, E.W., Linterman, M.A., and Fearon, D.T. (2014). Fibroblastic reticular cells of the lymph node are required for retention of resting but not activated CD8+ T cells. *Proc Natl Acad Sci U S A* 111**,** 12139-12144. doi: 10.1073/pnas.1412910111.

Dubrot, J., Duraes, F.V., Potin, L., Capotosti, F., Brighouse, D., Suter, T., Leibundgut-Landmann, S., Garbi, N., Reith, W., Swartz, M.A., and Hugues, S. (2014). Lymph node stromal cells acquire peptide-MHCII complexes from dendritic cells and induce antigen-specific CD4(+) T cell tolerance. *J Exp Med* 211**,** 1153-1166. doi: 10.1084/jem.20132000.

Gregg, R.K., Nichols, L., Chen, Y., Lu, B., and Engelhard, V.H. (2010). Mechanisms of spatial and temporal development of autoimmune vitiligo in tyrosinase-specific TCR transgenic mice. *J Immunol* 184**,** 1909-1917. doi: 10.4049/jimmunol.0902778.

Hammerschmidt, S.I., Ahrendt, M., Bode, U., Wahl, B., Kremmer, E., Forster, R., and Pabst, O. (2008). Stromal mesenteric lymph node cells are essential for the generation of gut-homing T cells in vivo. *J Exp Med* 205**,** 2483-2490. doi: 10.1084/jem.20080039.

Jang, J.Y., Koh, Y.J., Lee, S.H., Lee, J., Kim, K.H., Kim, D., Koh, G.Y., and Yoo, O.J. (2013). Conditional ablation of LYVE-1+ cells unveils defensive roles of lymphatic vessels in intestine and lymph nodes. *Blood* 122**,** 2151-2161. doi: 10.1182/blood-2013-01-478941.

Lee, J.W., Epardaud, M., Sun, J., Becker, J.E., Cheng, A.C., Yonekura, A.R., Heath, J.K., and Turley, S.J. (2007). Peripheral antigen display by lymph node stroma promotes T cell tolerance to intestinal self. *Nat Immunol* 8**,** 181-190. doi: 10.1038/ni1427.

Limmer, A., Ohl, J., Wingender, G., Berg, M., Jungerkes, F., Schumak, B., Djandji, D., Scholz, K., Klevenz, A., Hegenbarth, S., Momburg, F., Hammerling, G.J., Arnold, B., and Knolle, P.A. (2005). Cross-presentation of oral antigens by liver sinusoidal endothelial cells leads to CD8 T cell tolerance. *Eur J Immunol* 35**,** 2970-2981. doi: 10.1002/eji.200526034.

Lund, A.W., Duraes, F.V., Hirosue, S., Raghavan, V.R., Nembrini, C., Thomas, S.N., Issa, A., Hugues, S., and Swartz, M.A. (2012). VEGF-C promotes immune tolerance in B16 melanomas and cross-presentation of tumor antigen by lymph node lymphatics. *Cell Rep* 1**,** 191-199. doi: 10.1016/j.celrep.2012.01.005.

Mackay, F., and Browning, J.L. (1998). Turning off follicular dendritic cells. *Nature* 395**,** 26-27. doi: 10.1038/25630.

Magnusson, F.C., Liblau, R.S., Von Boehmer, H., Pittet, M.J., Lee, J.W., Turley, S.J., and Khazaie, K. (2008). Direct presentation of antigen by lymph node stromal cells protects against CD8 T-cell-mediated intestinal autoimmunity. *Gastroenterology* 134**,** 1028-1037. doi: 10.1053/j.gastro.2008.01.070.

Mcculloch, L., Brown, K.L., Bradford, B.M., Hopkins, J., Bailey, M., Rajewsky, K., Manson, J.C., and Mabbott, N.A. (2011). Follicular dendritic cell-specific prion protein (PrP) expression alone is sufficient to sustain prion infection in the spleen. *PLoS Pathog* 7**,** e1002402. doi: 10.1371/journal.ppat.1002402.

Nichols, L.A., Chen, Y., Colella, T.A., Bennett, C.L., Clausen, B.E., and Engelhard, V.H. (2007). Deletional self-tolerance to a melanocyte/melanoma antigen derived from tyrosinase is mediated by a radio-resistant cell in peripheral and mesenteric lymph nodes. *J Immunol* 179**,** 993-1003.

Onder, L., Scandella, E., Chai, Q., Firner, S., Mayer, C.T., Sparwasser, T., Thiel, V., Rulicke, T., and Ludewig, B. (2011). A novel bacterial artificial chromosome-transgenic podoplanin-cre mouse targets lymphoid organ stromal cells in vivo. *Front Immunol* 2**,** 50. doi: 10.3389/fimmu.2011.00050.

Rouhani, S.J., Eccles, J.D., Riccardi, P., Peske, J.D., Tewalt, E.F., Cohen, J.N., Liblau, R., Makinen, T., and Engelhard, V.H. (2015). Roles of lymphatic endothelial cells expressing peripheral tissue antigens in CD4 T-cell tolerance induction. *Nat Commun* 6**,** 6771. doi: 10.1038/ncomms7771.

Tamburini, B.A., Burchill, M.A., and Kedl, R.M. (2014). Antigen capture and archiving by lymphatic endothelial cells following vaccination or viral infection. *Nat Commun* 5**,** 3989. doi: 10.1038/ncomms4989.

Tewalt, E.F., Cohen, J.N., Rouhani, S.J., Guidi, C.J., Qiao, H., Fahl, S.P., Conaway, M.R., Bender, T.P., Tung, K.S., Vella, A.T., Adler, A.J., Chen, L., and Engelhard, V.H. (2012). Lymphatic endothelial cells induce tolerance via PD-L1 and lack of costimulation leading to high-level PD-1 expression on CD8 T cells. *Blood* 120**,** 4772-4782. doi: 10.1182/blood-2012-04-427013.

Wang, X., Cho, B., Suzuki, K., Xu, Y., Green, J.A., An, J., and Cyster, J.G. (2011). Follicular dendritic cells help establish follicle identity and promote B cell retention in germinal centers. *J Exp Med* 208**,** 2497-2510. doi: 10.1084/jem.20111449.

Yau, I.W., Cato, M.H., Jellusova, J., Hurtado De Mendoza, T., Brink, R., and Rickert, R.C. (2013). Censoring of self-reactive B cells by follicular dendritic cell-displayed self-antigen. *J Immunol* 191**,** 1082-1090. doi: 10.4049/jimmunol.1201569.
