# Supplementary material for: Modes of Antigen Presentation by Lymph Node Stromal Cells and Their Immunological Implications
Source: Front Immunol. 2015 Sep 8;6:446. doi: 10.3389/fimmu.2015.00446 (PMC4561840; doi:10.3389/fimmu.2015.00446)
Supplement: Supplementary file 2 [file Image_1.PDF]

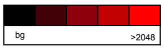

SCAVENGER RECEPTORS		alternative names	Known Ag Routing	FRC MLN	FRC SLN	LEC MLN	LEC SLN	BEC MLN	BEC SLN	DN
Class A	SR-AI	Msr1, Scara1, SRA, CD204								
	SR-AII	Msr1								
	CSR1	Scara3								
	MARCO	Scara 2								
	SCARA5									
	SRCL	Scara 4, Colec12								
Class B	CD36	Scarb3								
	SR-BI	Scarb1								
	LIMP2	Scarb2								
Class C*	dSR-CI									
Class D	CD68									
Class E	LOX-1	Olr1, Clec8a								
Class F	SREC-I	Scarf1								
	SREC-2	Scarf2								
Class G	SR-PSOX	Cxcl16								
Class H	FEEL-1	Stab1, CLEVER-1								
	FEEL-2	Stab2								
Class I	CD163									
	CD163L1									
	CD5	CD50, Icam5								
	CD6									

* only in drosophila

Fc RECEPTORS		alternative names	Known Ag Routing	FRC MLN	FRC SLN	LEC MLN	LEC SLN	BEC MLN	BEC SLN	DN
activating	Fcgr1									
inhibitory	Fcgr2b	FCR-2, CD32b								
activating	Fcgr3	CD16								
activating	FcRgamma	Fcer1g								
	FcRn	Fcgrt, FcR IgG α chain transporter								

COMPLEMENT RECEPTOR		alternative names	Known Ag Routing	FRC MLN	FRC SLN	LEC MLN	LEC SLN	BEC MLN	BEC SLN	DN
antigen recognition /uptake	CR1	CD35								
	CR2	CD21								
	CR3 - CD18	CD18/CD11b								
	CD11b									
	CR4	CD18/CD11c								
	C1qR	CD93								
Pathogen recognition/clearance	CRIg	Vsig4								
Modulation of Th1/2	C3aR									
	C5aR	CD88								
Cytokine modulation, APC maturation	CD46	MCP								
	CD55	DAF								
	CD59	MAC inhibitor								

From Kemper and Atkinson 2007 Nature Review Immunology

SOME KNOWN RECEPTORS DIRECTED TO ANTIGEN PROCESSING		alternative names	Known Ag Routing	FRC MLN	FRC SLN	LEC MLN	LEC SLN	BEC MLN	BEC SLN	DN
Mannose Receptor Family	CD206	Mrc1	MHC I							
	Endo180	Mrc2								
	PLA2R									
	CD205	DEC205, Ly75								
Siglecs (Ig-like lectins)	CD169	Siglec-I								
C-type lectins	CD207	Clec4k, langerin								
	CD209a	mouse DC-SIGN, SIGNR1, Clec4l								
	DCIR	Clec4a2, DC-inhibitory receptor 2								
	DNGR1	Clec9a	both MHC I and II							
	dectin-1	Clec7a, b-glucan receptor	both MHC I and II							
	dectin-2	Clec6a, Clec4n								
Complement regulatory protein	CD59	Cd59a	both MHC I and II							
LDL receptor Family	CD91	LRP1, ApoER, A2MR								

UPTAKE OF APOPTOTIC CELLS		alternative names	Known Ag Routing	FRC MLN	FRC SLN	LEC MLN	LEC SLN	BEC MLN	BEC SLN	DN
	CED-1	MEGF10								
	CED-2	CrkII								
	CED-5	Dock2								
	CED-6	GULP								
	CED-7	ABC transporter*								
	CED-10	Rac1								
	CED-12	ELMO1								

From Nagata Hanayama and Kawane 2010 Cell
* in C elegans

TLR				FRC MLN	FRC SLN	LEC MLN	LEC SLN	BEC MLN	BEC SLN	DN
	TLR1									
	TLR2									
	TLR3									
	TLR4									
	TLR5*									
	TLR6									
	TLR7									
	TLR8									
	TLR9									

* not in Immgen

Supplemental Figure 1. LNSC expression of receptors known to be relevant in antigen uptake and processing. Select gene expression levels of scavenger receptors, Fc receptors, complement receptors, known receptors for and those that influence antigen processing from the Immunological Genome Project are organized as a heatmap. The v1 resting stromal cell dataset was used.
