# Supplementary material for: Modes of Antigen Presentation by Lymph Node Stromal Cells and Their Immunological Implications
Source: Front Immunol. 2015 Sep 8;6:446. doi: 10.3389/fimmu.2015.00446 (PMC4561840; doi:10.3389/fimmu.2015.00446)
Supplement: Supplementary file 3 [file Image_2.PDF]

COSTIMULATORY AND COINHIBITORY RECEPTORS				FRC		LEC		BEC		DN
		alternative names	binding partners	MLN	SLN	MLN	SLN	MLN	SLN	
Costimulatory	4-1BBL	Tnsf9	4-1BB							
	OX40L	Tnsf4	OX40							
	CD70	Tnfsf7	CD27							
	CD80	B7-1	CD28, CTLA4							
	CD86	B7-2	CD28, CTLA4							
	CD40		CD40L							
	B7-H6		NCR3/NKp30							
	B7-H2	iCOS-L	iCOS, CD28, CTLA-4							
	GITRL	TNFSF18	GITR							
	ICAM-1		LFA-1 (CD18/CD11a)							
	ICAM-2	CD102	CD18							
	LFA-3		LFA-2(CD2)							
	LFA-1	DC-DESIGN, ITGAL	ICAM-3							
Coinhibitory	PD-L1	CD274, B7-H1	PD-1							
	PD-L2	Pdcd1lg2, B7-DC	PD-1							
	FasL		Fas							
	CD153	Tnfsf8	CD30							
	TRAIL	Tnfsf10	TRAIL-R							
	HVEM	Tnfrsf14	CD160, BTLA, 2B4, LIGHT							
	CD48		2B4							
	MHCII		LAG3							
	B7-H3	CD276	?							
	B7-H4	Vtcn1	?							

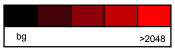

Supplemental Figure 2. LNSC expression of antigen processing genes. Select gene expression levels of proteins known to be involved in antigen processing pathways from the Immunological Genome Project are organized as a heatmap. The v1 resting stromal cell dataset was used.
