# Supplementary material for: Modes of Antigen Presentation by Lymph Node Stromal Cells and Their Immunological Implications
Source: Front Immunol. 2015 Sep 8;6:446. doi: 10.3389/fimmu.2015.00446 (PMC4561840; doi:10.3389/fimmu.2015.00446)
Supplement: Supplementary file 4 [file Image_3.PDF]

bg >2048

| ANTIGEN PRESENTATION | | | | FRC | | LEC | | BEC | | DN |
|---|---|---|---|---|---|---|---|---|---|---|
| | | alternative names | involved in | MLN | SLN | MLN | SLN | MLN | SLN | |
| MHC-I | Sec61 | Sec61a | Endosome to cytoplasm export | | | | | | | |
| | p97 | VCP, IBMPFD | | | | | | | | |
| | hsp90 | | | | | | | | | |
| | IRAP | Lnpep | Endosomal processing | | | | | | | |
| | TRIM21 | | Opsonized particle delivery to proteasome | | | | | | | |
| | LMP2 | Psmb9 | Cytoplasmic processing (immunoproteasome) | | | | | | | |
| | LMP7 | Psmb8 | | | | | | | | |
| | LMP10 | Psmb10, Mecl1 | | | | | | | | |
| | MB1 | Psmb5, X | Cytoplasmic processing (constitutive) | | | | | | | |
| | Z | Psmb7 | | | | | | | | |
| | delta | Psmb6, Y | | | | | | | | |
| | TAP1 | | ER import | | | | | | | |
| | TAP2 | | | | | | | | | |
| | tapasin | Tapbp | | | | | | | | |
| | ERAP1 | | ER processing | | | | | | | |
| | PLAP | Lnpep | | | | | | | | |
| | ERp57 | Pdia3 | | | | | | | | |
| | Glucosidase I | Gls | MHC-I maturation and stability | | | | | | | |
| | Glucosidase II | GlsII | | | | | | | | |
| | Calreticulin | Calr, CLT | | | | | | | | |
| | Calnexin | Canx, CLX | | | | | | | | |
| | B2M | | | | | | | | | |
| | UGT1 | | | | | | | | | |
| | Sec22b | | Phagosome ER fusion | | | | | | | |
| | Syntaxin 4 | Stx4a | | | | | | | | |
| | Syntaxin 18 | Stx18 | | | | | | | | |
| MHCII | LAMP1 | | Lysome membrane markers | | | | | | | |
| | LAMP2 | | | | | | | | | |
| | cathepsin Z | ctsz | Lysosomal processing | | | | | | | |
| | cathepsin H | ctsh | | | | | | | | |
| | cathepsin C | ctsc | | | | | | | | |
| | GILT | Ifi30 | | | | | | | | |
| | CIITA | | MHC-II stability | | | | | | | |
| | H2-M | H-2DMa | | | | | | | | |
| | H2-M | H-2DMb2 | | | | | | | | |
| | Ii | CD74, HLA-DG | | | | | | | | |
| | cathepsin S | ctss | | | | | | | | |
| | cathepsin L | ctsl | | | | | | | | |
| | H2-O | H2-Oa | | | | | | | | |
| | H2-O | H2-Ob | | | | | | | | |
| | MARCH1 | | | | | | | | | |
| | LC3a | Map1lc3a | Autophagy | | | | | | | |
| | LC3b | Map1lc3b | | | | | | | | |
| | Atg12 | | | | | | | | | |
| | Atg8 | | | | | | | | | |
| | Beclin1 | Becn1 | | | | | | | | |
| | Atg 5 | | | | | | | | | |
| | Atg 7 | | | | | | | | | |
| | Atg 16L1 | | | | | | | | | |

**Supplemental Figure 3.** LNSC expression of costimulatory and coinhibitory molecules. Select gene expression levels of costimulatory and coinhibitory molecules from the Immunological Genome Project are organized as a heatmap. The v1 resting stromal cell dataset was used.
